# Bubble thermodynamics in cryogenic fluids under ultrasonic field excitation: Theoretical analysis and numerical calculation

**DOI:** 10.1016/j.ultsonch.2024.106969

**Published:** 2024-06-22

**Authors:** Jin Zhang, Yu Zhang, Yong Chen, Xiaobo Rui, Yao Yu, Yu Wu, Jie Yang, Lei Qi

**Affiliations:** a State Key Laboratory of Precision Measurement Technology and Instruments, Tianjin University, Tianjin 300072, China; b Chengdu Fluid Dynamic Innovation Center, Chengdu 610071, China; c Beijing Institute of Spacecraft Environment Engineering, Beijing 100094, China

**Keywords:** Bubble thermodynamic, Heat transfer mechanism, Cryogenic fluids, Bubble–bubble interaction

## Abstract

In the study of cavitation in room-temperature fluids, the heat transfer between gas and liquid in bubble oscillation is usually assumed to be an adiabatic process for simplification. However, this heat transfer and thermodynamic mechanism is not yet understood in cryogenic fluids, especially under small amplitude oscillation conditions excited by ultrasonic field. This article studies bubble thermodynamic model under an external ultrasonic field based on the heat transfer equation for cryogenic fluids. The temperature changes inside bubbles are calculated, and the heat transfer mechanism is briefly analyzed. The results indicate that the heat transfer mechanism of bubbles depends on the relationship between ultrasonic frequency and bubble resonance frequency. By analyzing two special cases of dual-bubble and high-pressure environment, it is believed that heat transfer can be approximated as an adiabatic process under high-pressure conditions with ultrasonic frequency far from the resonance frequency. This conclusion can provide a theoretical basis for subsequent accurate calculation of heat-transfer polytropic coefficient, or void faction measurement in cryogenic two-phase flow.

## 1 Introduction

Cryogenic fluids are becoming increasingly widely used in industries and research fields such as aerospace, chemical engineering and cryogenic wind tunnels. Common cryogenic fluids include liquid nitrogen, liquid oxygen, liquid methane, etc. During the transmission and storage of cryogenic fluids, complex phase transitions and cavitation phenomena will occur due to factors such as wall heating, pressure changes, helium injection, resulting in the formation of bubbles of varying sizes dispersed in the liquid [1]. The study of bubble dynamic under ultrasonic field in cryogenic fluids is of great significance. For example, bubble oscillation mechanism induced by ultrasonic wave is one of the important problems in gas–liquid two phase flow measurement [2]. Current researches about bubble dynamic focus on the theoretical derivation and experimental observation of water in normal pressure and temperature, lacking researches in cryogenic fluids.

The basic method for bubble dynamic research is establishing governing equation based on continuity and momentum equation, while the solution of bubble governing equation is dependent on the pressure at bubble interface. Perform force analysis on oscillating bubble, considering surface tension and viscosity effect [3], the pressure on the interface can be approximately expressed as

(1) $$P_{B}=P_{\mathrm{in}}-\frac{2\sigma }{R_{b}}-\frac{4\mu_{l}\dot{R}}{R_{b}},$$

where *P_in_* is the total pressure inside bubble, *σ* is surface tension coefficient, *μ_l_* is liquid viscosity coefficient, *R_b_* is radium and $\dot{R}$ is the bubble wall velocity. Surface tension and viscous forces are usually considered as components of *P_in_* [4]. For *P_in_*_,_ existing research usually assumed that the gas is ideal gas, or calculated it as the superposition of two parts [5]:

(2) $$P_{\mathrm{in}}=P_{g}+P_{v},$$

where *P_g_* represents the ideal gas pressure, and *P_v_* represents the liquid vapour pressure. *P_v_* is usually assumed constant, while *P_g_* can be expressed by the equation of state of ideal gas in polytropic form *pV^κ^* = constant, *V* is the volume of bubble. Under this circumstance, *P_g_* is

(3) $$P_{g}=P_{g0}{\left(\frac{R_{0}}{R_{b}}\right)}^{3\kappa },$$

(4) $$P_{g0}=P_{l}+\frac{2\sigma }{R_{0}}-P_{v0}.$$

The subscript 0 means initial value, *P_l_* is the liquid pressure at infinity. Obviously bubble oscillation is an isothermal process when *κ* = 1, while most researches replace *κ* by the ratio of specific heats *γ* [6], which represents adiabatic process. Based on this assumption, Minnaert frequency is proposed to describe the bubble resonance frequency by linearizing the bubble governing equation:

(5) $$f_{\mathrm{res}}=\frac{1}{2\pi }\sqrt{\frac{1}{\rho_{l}R_{0}^{2}}\left(3\gamma P_{l}+\frac{2\sigma }{R_{0}}\left(3\gamma -1\right)\right)},$$

*ρ_l_* in formula (5) represents liquid density. Formulas (1)-(5) have been applied to most bubble dynamic researches without heat transfer and phase change [7].

However, polytropic coefficient cannot accurately describe the heat transfer process in bubble oscillation. The value of *κ* exists in the interval of 1 to *γ*, depending on the effective thermal damping caused by temperature change and energy loss of gas. Therefore, *κ* varies in different scenarios, such as large/small amplitude oscillations, pool boiling phenomenon, and so on. Prosperetti conducted series of researches on the precise solution of this polytropic constant. He firstly analyzed *κ* under acoustic cavitation [8], then proposed that as the frequency of acoustic wave increases from low to high, the heat transfer of bubble performs transitions from isothermal state to adiabatic state. As the frequency continues to increase, the value of *κ* could deviate from the range of 1 to *γ*. Properetti [9], [10] further studied the effect of heat transfer on bubble oscillation and provided an approximate calculation formula based on the equation of energy:

(6) $$\kappa =\frac{1}{3}\text{Re}\left(\varphi \right),$$

(7) φ=3γ1-3γ-1iχi/χ12cothi/χ12-1,χ=DωR02,

where *D* is the thermal diffusivity in the liquid, *ω* is angular frequency of ultrasonic wave. It should be noted that this relationship holds only when the displacement *X* of bubble wall has the same dependence on the acoustic field in time. The resonance frequency related to *κ* can be calculated from formula (5) by substituting *κ* into *γ*. This conclusion has been applied in many two-phase flow void fraction measurement and sound propagation studies [11], [12].

Except for polytropic coefficient, a more applicable method is calculating from equation of state *p* = *ρRT*. Assuming temperature is equal everywhere in gas and liquid at initial time leads to

(8) $$P_{g}=P_{g0}\left(\frac{T_{B}}{T_{0}}\right){\left(\frac{R_{0}}{R_{b}}\right)}^{3}.$$

*T_g_* and *T*_0_ are the average temperature and initial temperature of gas inside bubble, respectively. The thermodynamic problem caused by change of *T_B_* is the key to solve bubble oscillation process, making it necessary to introduce energy equation into governing equation.

The study of temperature changes inside bubbles firstly appeared in the 1960 s, focusing on cavitation or bubble generation scenarios. From perspective of energy equation, Flynn [13], [14] derived the bubble dynamic equation under the assumption of compressible fluid, and cited the transformation method proposed by Plesset and Zwick to obtain a differential equation system for calculating the temperature change of ideal gas inside the bubble. On the basis of Flynn, Fujikawa et al. [15] considered influence of phase transition in pool boiling of water on bubble dynamics. The gas inside bubble was divided into ideal gas(air) and water vapour in boiling heat transfer. Fujikawa used the Clausius-Clapeyron equation to calculate saturation vapour pressure, deeply discussed the changes in internal temperature and pressure during the process of bubble generation and collapse through numerical analysis.

Plesset and Zwick studied the heat transfer problem involved in generating spherical bubbles in liquids, and provided an approximate expression for gas temperature changes:

(9) $$T_{\infty }-T_{B}\left(t\right)=\frac{L_{l}\rho_{g}}{\rho_{l}C_{\mathrm{pl}}{\left(D_{l}\right)}^{\frac{1}{2}}}{\left(\frac{1}{\pi }\right)}^{\frac{1}{2}}\int_{0}^{t}\frac{R_{b}^{2}\left(x\right)\left(\frac{dR_{b}}{\mathrm{dt}}\right)dx}{{\left\{\int_{x}^{t}{\left[R_{b}\left(y\right)\right]}^{4}dy\right\}}^{\frac{1}{2}}},$$

where *T*_∞_ is liquid temperature at infinity area, *L_l_* represents liquid latent heat, while *C_pl_* is liquid specific heat at constant pressure. Based on the heat balance across bubble interface, Brennen [16] proposed a more proper parameter Σ, which is used as a term in Rayleigh-Plesset equation, to evaluate thermodynamic effects during bubble growth, which is defined by

(10) $$\Sigma =\frac{\rho_{v}L_{\mathrm{ev}}}{\rho_{l}^{2}C_{\mathrm{pl}}\sqrt{a_{l}}}\frac{dP_{v}}{\mathrm{dT}}.$$

Jean-Pierre Franc et al. [17], [18] further conducted theoretical and experimental researches to analyze the modifications of cavitation instabilities. Based on this theory, Dular et al. [3] calculated the temperature variations in liquid surrounding bubble, studied the thermodynamic effects caused by vapour pressure changes during the growth and collapse of a single cavitation bubble, and conducted experimental verification. Similar heat-quantity based method [19], [20], [21], [22] was used in series of studies to estimate temperature and mass change during bubble growth, with varying forms depending on different scenes.

There are also some other methods proposed. For example, Wolfgang Dreyer et al. [23], [24] used specific free energy to study the influence of the three phenomena heat conduction, elastic waves and phase transition on the evolution of the bubble. Qidong Yu et al. [25] investigated the thermodynamic effect during bubble collapse near a rigid boundary through numerical simulation. Thanh-Hoang Phan et al. [26] used experimental and numerical methods to explore the thermodynamic effects on single cavitation bubble dynamics under various ambient temperature conditions. What’s more, by summarizing room-temperature fluid researches, it can be found that cavitation research usually considers bubbles as a “vapour cavity” composed of ideal gases and liquid vapour inside. Some articles specifically study the related issues of ‘gas-vapour’ bubbles, such as Prosperetti’s group researched the dynamic model, linearization characteristics and sound wave propagation considering vapour inside bubble [27], [28], [29]. Xiaoyu Wang et al. [30] combined Laplace transform method with theory proposed in Ref. [28] to obtain the analytical solution of the non-dimensional perturbation of the instantaneous bubble radius during the transient process in the initial oscillation stage. Mirko Gallo et al. [31] presented a thorough model based on an isothermal diffuse interface description of a two-phase liquid–vapour system endowed with thermal fluctuations, exploiting fluctuating hydrodynamic theory.

In addition to the heat transfer mechanism, another difficulty in bubble measurement is that there are usually a large number of bubbles in practical bubble detection applications. Interactions between bubbles make it difficult for a single bubble model to accurately describe the dynamic characteristics of multiple bubbles [32]. The study of multi-bubble is mainly based on the second Bjerknes force formed by secondary radiation between bubbles, including multi-bubble dynamics [33], [34], [35], [36] and sound wave propagation in bubbly liquids [37], [11]. Similar to single bubble, most of these studies adopt the adiabatic assumption and use polytropic coefficients *γ* to represent the heat transfer process of bubbles. Due to the fact that the Bjerknes force on bubbles depends on factors such as the number of bubbles and bubble spacing, its heat transfer mechanism is more difficult to predict compared to a single bubble.

In terms of cryogenic fluids, the most common approach to thermodynamic and bubble problem is cavitation research. These researches usually divided cryogenic fluids into two parts: liquid and vapour, with a thermal-boundary layer and liquid–vapour interface between them. Early studies regarded cryogenic cavitation as a special case of room temperature cavitation, however the significant differences in thermophysical properties made this assumption unreasonable [38]. B-factor [39], [40] is firstly formulated to evaluate the ratio of vapour phase to liquid phase, including Gelder-Moore-Ruggeri method, MTWO method, entrainment theory, and Fruman method, etc. Brennen’s theory was also used in some Rayleigh-Plesset equation based methods [41], [42], as the thermodynamic parameter Σ avoids the limitation of B-factor by considering time and temperature effects [43]. Kyuichi Yasui [44] introduced the effect of the evaporation and condensation of nitrogen vapour into motion rate at the bubble wall, performed simulation research of bubble oscillations induced by an ultrasonic wave in liquid nitrogen. In recent years, Tianwei Lai et al. [45] developed a single bubble growth model in liquid hydrogen considering temperature distribution inside bubble. Raj Sarath [46] studied the bubble dynamics on thermal destratification and quantify the extent of destratification in cylindrical liquid hydrogen storage tanks. The liquid–vapour condensation and evaporation rate were usually adopted in these researches, as an evaluation of thermal effects and mass transfer. Due to the difficulty of conducting cryogenic fluid experiments, theoretical prediction on thermodynamic in cryogenic fluids is usually assisted by the superheat boiling of water. Sato et al. [47], [48] performed several experimental studies on the laser-induced bubble dynamics in liquid nitrogen, and some other experiments [49] shows that bubble dynamics in cryogenic fluids has significant difference compared with water.

In summary, the assumption of “ideal gas and vapour” is reasonable when conducting bubble thermodynamic research in cryogenic fluids. The relationship between saturated vapour pressure and temperature, density of fluid vapour cannot be described by formula (3) or (7). Some bubble cavitation studies or linearization analyses that consider fluid vapour also regard it as ideal gas and cannot fully conform to the characteristics of cryogenic fluids. This article assumed that the cryogenic fluid has weak compressibility, introduced equation of state in cryogenic fluids based on Helmholtz free energy and vapour pressure ancillary function into bubble governing equation, studied the thermodynamic characteristics of bubble oscillation under ultrasonic fields, analyzed the thermodynamic mechanism of bubbles through theoretical analysis and numerical calculations based on energy equations. Special conditions of dual-bubble and high pressure environment were calculated and analyzed in the article. Our research is based on the following assumptions:

(1) The gas inside bubble is composed of helium gas and liquid vapour in cryogenic fluids;
(2) The temperature change inside bubble is only caused by pressure changes;
(3) Bubble oscillation time under ultrasonic field is extremely short so there’s no thermal boundary layer formed;
(4) The liquid around the bubble has weak compressibility;
(5) The amplitude of bubble oscillation is small so both liquid and gas haven’t reached the critical point for phase change in cryogenic fluids.

The remainder of this paper is organized as follows. The bubble oscillation processes are derived from state equation of gas, bubble governing equation, and energy equation on liquid–gas interface in Section 2. Section 3 conducts numerical calculations and analyses for liquid oxygen and liquid methane respectively. The influence of ultrasonic frequency, bubble–bubble interaction, and high-pressure environment on bubble oscillation and heat transfer mechanism was discussed. Finally, conclusion is given in Section 4.

## 2 Method

The research on bubble thermodynamic is usually based on several key issues: governing equation derived from mass and momentum conservation equation, equation of state, and heat transfer equation, which jointly constitute the oscillation process of bubble. This section consists of four parts, namely: the state of equation for gas, bubble governing equation, the approximate solution of temperature change inside bubble and temperature change of liquid at interface.

### 2.1 Equation of state in cryogenic fluid

Room-temperature research typically adopts the ideal gas assumption. For gas in cryogenic fluids, fundamental equation of state could calculate complex pressure expressions, including both state and heat information [50]. Therefore, we can obtain thermodynamic properties through mathematical differentiation from fundamental equations. The most common method currently is using Helmholtz free energy [51], whose equation form is as follows:

(11) $$\alpha \left(\delta ,\tau_{g}\right)=\alpha^{0}\left(\delta ,\tau_{g}\right)+\alpha^{\text{r}}\left(\delta ,\tau_{g}\right),$$

*α*, *δ* and *τ_g_* are dimensionless parameters, representing Helmholtz free energy, density and temperature respectively, defined by

(12) $$\alpha =\frac{a}{R_{g}T_{g}},\delta =\frac{\rho_{g}}{\rho_{c}},\tau_{g}=\frac{T_{c}}{T_{g}}.$$

*R_g_* is gas constant, *ρ_g_* and *T_g_* are gas density and temperature, while *ρ_c_* and *T_c_* are critical points. *α*^0^ and *α^r^* are ideal gas and fluid compressibility contribution to Helmholtz free energy. The gas pressure in Helmholtz equation of state is

(13) $$\frac{P}{P_{c}}=\frac{\delta }{\tau_{g}Z_{c}}\left[1+\delta \left(\frac{\partial \alpha^{\text{r}}}{\partial \delta }\right)\right].$$

*Z_c_* is the critical compressibility factor and *P_c_* represents critical pressure. The compressibility factor is defined as

(14) $$Z=\frac{P_{g}}{\rho_{g}R_{g}T_{g}}=1+\delta \left(\frac{\partial \alpha^{\text{r}}}{\partial \delta }\right).$$

Substituting formula (14) to (13) yields

(15) $$P=\frac{\delta Z}{\tau Z_{c}}P_{c}.$$

According to formula (15), the component of ideal gas can be expressed as

(16) $$P_{i}=\frac{\delta_{i}}{\tau_{i}Z_{c}}P_{c}=\rho_{g}R_{g}T_{g},$$

(17) $$\frac{P_{i}}{P_{i0}}=\frac{\rho_{g}T_{g}}{\rho_{g0}T_{g0}}.$$

So we can divide formula (13) into two contributions similar to Helmholtz free energy: ideal gas and compressibility component. The ideal gas contribution is equal to the normal equation of state for ideal gas, for which we can adopt formula (8):

(18) $$P_{g}=P_{g0}\left(\frac{T_{B}}{T_{0}}\right){\left(\frac{R_{0}}{R_{b}}\right)}^{3}.$$

Due to the fact that there is no external heat inflow, the order of magnitude of temperature change inside the bubble is relatively low. According to the measurement and calculation of gas compressibility coefficient, the order of magnitude of pressure changes caused by it will be much lower than ideal gas pressure (see Appendix A), the gas inside the bubble can be approximated as ideal gas.

Assuming that bubble is composed of ideal gas and cryogenic liquid vapour, the saturated vapour pressure is given by [51]

(19) $$\ln\left(\frac{P_{v}}{P_{c}}\right)=\tau_{g}\sum_{i=1}^{q}N_{i}\theta^{k_{i}},\theta =1-\frac{1}{\tau_{g}}.$$

Formula (19) is an auxiliary function given by numerical fitting. Coefficients *N_i_*, exponents *k_i_* and *q* are constants, *θ* is a dimensionless temperature variable, so the saturated vapour pressure is only related to temperature. Write it as follow

(20) $$P_{v}=P_{c}e^{\tau_{g}\sum_{i=1}^{q}N_{i}\theta^{k_{i}}},$$

gas pressure (8) and saturation vapour pressure (20) constitute pressure inside bubble together.

### 2.2 Heat transfer and temperature change inside bubble

In this article, we considered the bubble governing equation and energy equation as dependent problems. Let *T_B_*(*R*,*t*) be the temperature inside bubble and *T_l_*(*R*,*t*) be the temperature in the liquid. The two temperature satisfy boundary and initial conditions at interface:

temperature continuous:

(21) $$T_{B}\left(R_{b},t\right)=T_{l}\left(R_{b},t\right)=T\left(t\right),$$

heat flux continuous:

(22) $$k_{g}{\left(\frac{\partial T_{B}}{\partial r}\right)}_{R_{b}}=k_{l}{\left(\frac{\partial T_{l}}{\partial r}\right)}_{R_{b}},$$

temperature is the same everywhere initially:

(23) $$T_{B}\left(r,0\right)=T_{l}\left(r,0\right)=T_{n},$$

and the temperature in liquid at infinity remains unchanged:

(24) $$\lim_{r\to \infty }T_{l}\left(r,t\right)=T_{n}.$$

*k_g_* and *k_l_* in formula (22) are thermal conductivity of gas and liquid respectively, *r* represents the distance between the points inside the bubble and the center of the bubble.

Under above conditions, the energy equation of gas inside gas–liquid interface is

(25) $$\rho C_{v}\left(\frac{\partial T}{\partial t}+u\frac{\partial T}{\partial r}\right)=k\nabla^{2}T-p\nabla \cdot u,$$

*u* means radial velocity of interface, and *C_v_* is specific heat at constant volume. By introducing transformation method $m=3\int_{0}^{r}\rho \left(\xi ,t\right)\xi^{2}d\xi$ and concept of temperature potential $\partial \phi /\partial m=T_{B}-T_{l}$, this equation can be rewritten as[15]

(26) $$\frac{\partial \phi }{\partial t}=9\frac{k_{g}}{C_{\mathrm{vg}}}\frac{r^{4}}{{\left(\rho_{g}R_{b}^{3}\right)}^{2}}\frac{\partial^{2}\phi }{\partial m^{2}}-\frac{3}{C_{\mathrm{vg}}R_{b}^{3}}\left[pr^{2}u-\int_{0}^{m}r^{2}u\frac{\partial p}{\partial m^{\prime }}dm^{\prime }\right].$$

Assuming that the pressure gradient inside the bubble is zero, and defining a new independent variable *τ* by $\tau =9\rho_{g}\int_{0}^{t}k_{g}\left(\xi^{\prime }\right)R\left(\xi^{\prime }\right)/C_{\mathrm{vg}}\left(\xi^{\prime }\right)d\xi^{\prime }$, yield

(27) $$\frac{\partial \phi }{\partial \tau }=\frac{\partial^{2}\phi }{\partial m^{2}}-\frac{3}{C_{\mathrm{vg}}}pr^{2}u.$$

Taking the assumption that the velocity within the cavity satisfies

(28) $$r^{2}u=\frac{r^{3}}{R_{b}}\dot{R}=\frac{mR_{b}^{2}}{\rho_{g}}\dot{R},$$

equation (27) can be transformed to

(29) $$\frac{\partial \phi }{\partial \tau }=\frac{\partial^{2}\phi }{\partial m^{2}}-\frac{3}{C_{\mathrm{vg}}\rho_{g}R_{b}}p\dot{R}m.$$

Equation (29) can be furthermore reduced to

(30) $$\frac{\partial W}{\partial \tau }=\frac{\partial^{2}W}{\partial m^{2}}$$

with

(31) $$W\left(m,\tau \right)=\phi \left(m,\tau \right)+\frac{3m}{\rho_{g}}\int_{0}^{\tau }\frac{p_{c}\left(\xi \right)\dot{R}\left(\xi \right)d\xi }{C_{\mathrm{vg}}R_{b}\left(\xi \right)}.$$

According to the continuity of temperature at interface, we have

(32) $$W\left(m,0\right)=0,W\left(0,\tau \right)=0,$$

and when *m* = 1, there is

(33) $${\left[\frac{\partial W}{\partial m}\right]}_{m=1}=T\left(\tau \right)+\frac{3m}{\rho_{g}}\int_{0}^{\tau }\frac{p_{c}\left(\xi \right)\dot{R}\left(\xi \right)d\xi }{C_{\mathrm{vg}}R_{b}\left(\xi \right)}-1=\psi_{0}\left(\tau \right).$$

Based on boundary conditions (21), (22), the general solution of equation (33) can be obtained through Laplace transform [52]:

(34) $$W\left(m,\tau \right)=\int_{0}^{\tau }\theta_{1}\left[\frac{m}{2}|i\pi \xi \right]\psi_{0}\left(\tau -\xi \right)d\xi ,$$

*θ*_1_ is theta function of first kind defined by

(35) $$\theta_{1}\left[\frac{m}{2}|i\pi \xi \right]=i\sum_{n=-\infty }^{\infty }{\left(-1\right)}^{n}\exp\left[-\pi^{2}{\left(n-\frac{1}{2}\right)}^{2}\tau +i\pi \left(n-\frac{1}{2}\right)m\right].$$

An auxiliary function could be used as approximation for *θ*_1_:

(36) $$h\left(m,\tau \right)=h_{0}\left(m,\tau \right)-h_{1}\left(m,\tau \right)+\cdots +{\left(-1\right)}^{k}h_{k}\left(m,\tau \right),$$

where *h_k_* corresponds to

(37) $$h_{k}\left(m,\tau \right)=\frac{C_{\mathrm{vg}}\sin\left\{\left[2k+1\right]\frac{\pi m}{2}\right\}}{3}\int_{0}^{\tau }e^{-{\left(2k+1\right)}^{2}\pi^{2}\frac{\xi }{4}}\frac{d\psi_{0}\left(\tau -\xi \right)}{d\left(\tau -\xi \right)}d\xi .$$

Transforming the integral in formula (36) by a change variable *χ* = *τ* − *ξ* yields

(38) $$h_{k}\left(m,\tau \right)=\frac{C_{\mathrm{vg}}\sin\left\{\left[2k+1\right]\frac{\pi m}{2}\right\}}{3}H_{k}\left(\tau \right).$$

*H_k_*(*τ*) satisfies the relation

(39) $$e^{-{\left(2k+1\right)}^{2}\pi^{2}\frac{\tau }{4}}H_{k}\left(\tau \right)=\frac{C_{\mathrm{vg}}}{3}\int_{0}^{\tau }e^{{\left(2k+1\right)}^{2}\pi^{2}\frac{\chi }{4}}d\chi ,$$

and it can be expressed as a differential equation

(40) $$\frac{dH_{k}}{d\tau }+\frac{{\left(2k+1\right)}^{2}\pi^{2}}{4}H_{k}=\frac{C_{\mathrm{vg}}}{3}\frac{d\psi_{0}}{d\tau }.$$

According to the definition of $\psi_{0}$ and *τ*, equation (40) finally transforms to

(41) $$\frac{dH_{k}}{\mathrm{dt}}+\left[\frac{9{\left(2k+1\right)}^{2}\pi^{2}\rho_{g}k_{g}}{4C_{\mathrm{vg}}}\right]R_{b}H_{k}=\frac{P_{c}}{\rho_{g}R_{b}}\dot{R}+\frac{C_{\mathrm{vg}}}{3}\frac{dT_{B}}{\mathrm{dt}},$$

For the convenience of calculation, we take variables *R*,$\dot{R}$ and *T_B_* as functions with dependent variable *t*. Therefore, the general solution of equation (41) is given by (see Appendix B)

(42) $$H_{k}\left(t\right)=e^{-\int M_{k}\left(t\right)dt}\cdot \left(\int N_{k}\left(t\right)e^{\int M_{k}\left(t\right)dt}dt+C\right),$$

where *C* is constant, *M_k_*(*t*) and *N_k_*(*t*) are defined from equation:

(43) $$M_{k}\left(t\right)=\left[\frac{9{\left(2k+1\right)}^{2}\pi^{2}\rho_{g}k_{g}}{4C_{\mathrm{vg}}}\right]R_{b},N_{k}\left(t\right)=\frac{P_{c}}{\rho_{g}R_{b}}\dot{R}+\frac{C_{\mathrm{vg}}}{3}\frac{dT_{B}}{\mathrm{dt}}.$$

There exists *H_k_*(0) = 0 due to temperature being equal everywhere at initial time. Substituting it into formula (42) shows that *C* = 0, so

(44) $$H_{k}\left(t\right)=e^{-\int M_{k}\left(t\right)dt}\cdot \int N_{k}\left(t\right)e^{\int M_{k}\left(t\right)dt}dt.$$

Formula (43) and (44) constitute the solution of equation (41). The physical meaning of *H_k_* is the ratio of heat *Q* to mass *m*, so the gradient of *T_B_* is calculated according to formula (37):

(45) $$\frac{\partial T_{B}}{\partial m}=\frac{6}{C_{\mathrm{vg}}}h\left(m,\tau \right)=\frac{6}{C_{\mathrm{vg}}}\sum_{k=0}^{\infty }{\left(-1\right)}^{k}H_{k}\left(\tau \right)\sin\left[\frac{1}{2}\left(2k+1\right)\pi m\right].$$

Integrating formula (45) and transforming *τ* to *t* get

(46) $$T_{B}\left(m,t\right)=T\left(t\right)+\sum_{k=0}^{\infty }{\left(-1\right)}^{k+1}\frac{12}{\left(2k+1\right)C_{\mathrm{vg}}\pi }H_{k}\left(t\right)\sin\left[\frac{1}{2}\left(2k+1\right)\pi m\right].$$

In particular, the temperature gradient at interface is

(47) $${\left(\frac{\partial T_{B}}{\partial r}\right)}_{r=R}=\frac{18\rho_{g}H}{C_{\mathrm{vg}}R_{b}},$$

and the heat transported across the interface is

(48) $$\frac{\mathrm{dQ}}{\mathrm{dt}}=4\pi k_{g}R_{b}^{2}{\left(\frac{\partial T_{B}}{\partial r}\right)}_{r=R}=\frac{72\pi \rho_{g}k_{g}R_{b}H}{C_{\mathrm{vg}}}.$$

So the average temperature inside bubble *T_B_*(*t*) is

(49) $$\begin{array}{l} T_{B}\left(t\right)=\frac{3}{R_{b}^{3}}\int_{0}^{R_{b}}T_{B}\left(m,t\right)r^{2}dr\\ \;\;=T_{l}\left(t\right)-\frac{3}{R_{b}^{3}}\int_{0}^{R_{b}}r^{2}dr\sum_{k=0}^{\infty }{\left(-1\right)}^{k+1}\frac{12}{\left(2k+1\right)C_{\mathrm{vg}}\pi }H_{k}\left(t\right)\sin\left[\frac{1}{2}\left(2k+1\right)\pi m\right]. \end{array}$$

By the definition of *T_B_*(*m*, *t*), formula (49) can be simplified to

(50) $$T_{B}\left(t\right)=T_{l}\left(t\right)-G\left(t\right)/T_{B}\left(t\right),$$

with

(51) $$G\left(t\right)=\frac{2}{\pi \rho_{g}}\left(g_{0}+\frac{g_{1}}{3}+\frac{g_{2}}{5}+\frac{g_{3}}{7}\right)T_{l}\left(t\right)-\frac{1}{2\rho_{g}}\left(g_{0}^{2}+g_{1}^{2}+g_{2}^{2}+g_{3}^{2}\right),$$

(52) $$g_{k}=\frac{12}{\left(2k+1\right)C_{\mathrm{vg}}\pi }H_{k}.$$

Finally, the relationship between *T_B_*, *T_l_* and *G* is

(53) $$T_{B}\left(t\right)=\frac{1}{2}\left[T_{l}\left(t\right)+{\left(T_{l}{\left(t\right)}^{2}-4G\left(t\right)\right)}^{0.5}\right].$$

### 2.3 Temperature change of the liquid at interface

The energy equation for liquid outside bubble in spherically symmetrical motion is

(54) $$\left(\frac{\partial T_{l}}{\partial t}+u\frac{\partial T_{l}}{\partial r}\right)=\frac{k_{l}}{\rho_{l}C_{\mathrm{vl}}}\frac{1}{r^{2}}\frac{\partial }{\partial r}\left[r^{2}\frac{\partial T_{l}}{\partial r}\right].$$

Taking the material coordinates similar to gas:$m_{l}=1+\rho_{l}\left(r^{3}-R_{b}^{3}\right)$ and $\tau =9\rho_{l}^{2}k_{l}/C_{\mathrm{vl}}\int_{0}^{t}R_{b}^{4}\left(\xi \right)d\xi$, so the energy equation transformed into

(55) $$\frac{\partial T_{l}}{\partial \tau_{l}}=\frac{\partial^{2}T_{l}}{\partial m_{l}^{2}}.$$

According to the continuity of temperature and heat flux at interface, we have

(56) $$T_{l}\left(1,\tau_{l}\right)=T_{B}\left(1,\tau_{g}\right).$$

(57) $${\left[\frac{\partial T_{l}}{\partial r}\right]}_{R_{b}}=\frac{k_{g}}{k_{l}}{\left[\frac{\partial T_{g}}{\partial r}\right]}_{R_{b}}.$$

Formula (57) can also be expressed by

(58) $${\left[\frac{\partial T_{l}}{\partial m_{l}}\right]}_{m_{l}=1}={\left(\frac{k_{g}}{k_{l}}\right)}^{2}\left(\frac{\rho_{g}}{\rho_{l}}\right){\left[\frac{\partial T_{g}}{\partial m_{g}}\right]}_{m_{l}=1}.$$

For temperature in liquid, we can use Green function to solve this equation[15]

(59) $$\begin{array}{c} T_{l}\left(m_{l},\tau_{l}\right)=-\frac{1}{4\pi }\int_{0}^{\tau_{l}}{\left[\frac{\partial T_{l}}{\partial m_{l}^{\prime }}\right]}_{m_{l}=1}G\left(m_{l},\tau_{l}|1,\tau_{l}^{\prime }\right)d\tau_{l}^{\prime }\\ +\frac{1}{4\pi }\int_{1}^{\infty }T_{l}\left(m_{l}^{\prime },0\right)G\left(m_{l},\tau_{l}|m_{l}^{\prime },0\right)dm_{l}^{\prime }, \end{array}$$

where

(60) $$G\left(m_{l},\tau_{l}|m_{l}^{\prime },\tau_{l}^{\prime }\right)=2\left(\frac{\pi }{\tau_{l}-\tau_{l}^{\prime }}\right)\left(\exp\left[\frac{-{\left(m_{l}+m_{l}^{\prime }-2\right)}^{2}}{4\left(\tau_{l}-\tau_{l}^{\prime }\right)}\right]+\exp\left[\frac{{\left(m_{l}-m_{l}^{\prime }\right)}^{2}}{4\left(\tau_{l}-\tau_{l}^{\prime }\right)}\right]\right),$$

*m_l_* can be set to 1 since only the liquid temperature needs to be calculated here. The simultaneous (59) and (60) finally obtain

(61) $$T_{l}\left(t\right)=1+\int_{0}^{\tau \left(t\right)}\frac{F\left(\varepsilon \right)d\left(\tau \left(\varepsilon \right)\right)}{{\left[\tau \left(t\right)-\tau \left(\varepsilon \right)\right]}^{0.5}},$$

formula (61) is the result of transforming *τ_l_*(*t*) into *t*. *F* is defined by

(62) $$F\left(t\right)=-\frac{1}{\sqrt{\pi }}{\left(\frac{k_{g}}{k_{l}}\right)}^{2}\left(\frac{\rho_{g}}{\rho_{l}}\right){\left[\frac{\partial T}{\partial m}\right]}_{m=1}=-\frac{6}{\sqrt{\pi }}\left(\frac{k_{g}}{k_{l}}\right)\left(\frac{\rho_{g}}{\rho_{l}}\right)\frac{1}{C_{\mathrm{vg}}}\frac{H\left(t\right)}{R_{b}^{3}},$$

and *τ_l_*(*t*) is

(63) $$\tau_{l}\left(t\right)=9\rho_{l}^{2}\left(\frac{k_{l}}{C_{\mathrm{vl}}}\right)\int_{0}^{t}R_{b}^{4}\left(\varepsilon \right)d\varepsilon ,$$

formulas (61), (62) and (63) constitute the calculation method for *T_l_*. By combining formulas (43), (44), (51–53) and (61–63), we can get average temperature *T_B_* inside bubble.

### 2.4 Bubble governing equation and numerical solution

In the study of bubble dynamics in room-temperature fluids, it is common to adopt the incompressible assumption. The bubble governing equations for incompressible liquids are expressed as[2]:

(64) $$R_{b}\ddot{R}+\frac{3}{2}{\dot{R}}^{2}=\frac{1}{\rho_{l}}\left(P_{B}-P\right),$$

*P_B_* is calculated by formula (1), (2), (8), (15), *T_B_* in formula (8) is calculated by the method in subsections 2.2 and 2.3. *P* represents the environmental pressure, defined as

(65) $$P=P_{l}-P_{a},$$

*P_l_* is the initial pressure of liquid, and *P_a_* is the pressure disturbance on the bubble surface. In single bubble oscillation, the pressure change on the bubble surface comes from the acoustic pressure, so *P_a_* is

(66) $$P_{a}=\varepsilon \exp\left(i\omega t-kx\right).$$

Cryogenic fluids usually have weak compressibility, and the bubble governing equation considering the compressibility of fluid is[9]

(67) $$R_{b}\ddot{R}\left(1-\frac{\dot{R}}{c_{l}}\right)+\frac{3}{2}{\dot{R}}^{2}\left(1-\frac{\dot{R}}{3c_{l}}\right)=\frac{1}{\rho_{l}}\left(1+\frac{\dot{R}}{c_{l}}+\frac{R_{b}}{c_{l}}\frac{d}{\mathrm{dt}}\right)\left(P_{B}-P\right),$$

where *c_l_* is sound speed in liuqid, the calculation methods for *P_B_* and *P* are the same as those under compressible assumption.

Expanding a single bubble into dual-bubble system results in the bubbles simultaneously being subjected to the interaction forces between bubbles caused by oscillation. Based on the second Bjerknes force, the pressure disturbance on the outer side of bubble wall is[53]

(68) $$P_{a,1}=\varepsilon \exp\left(i\omega t-kx\right)+\rho_{l}{\left(\frac{2R_{2}{\dot{R}}_{2}+R_{2}^{2}{\ddot{R}}_{2}}{d-R_{1}-R_{2}}\right)}_{t-(d-R_{1}-R_{2})/c}$$

and

(69) $$P_{a,2}=\varepsilon \exp\left(i\omega t-kx\right)+\rho_{l}{\left(\frac{2R_{1}{\dot{R}}_{1}+R_{1}^{2}{\ddot{R}}_{1}}{d-R_{1}-R_{2}}\right)}_{t-(d-R_{1}-R_{2})/c},$$

*R*_1_, *R*_2_ are radius of dual-bubbles.

The numerical solution is calculated by the forth-order Runge-Kutta method based on equation (67), which can be rewritten as

(70) $$\ddot{R}=\frac{\frac{1}{\rho_{l}}\left(1+\frac{\dot{R}}{c}+\frac{R_{b}}{c}\frac{d}{\mathrm{dt}}\right)\left(P_{B}-P\right)-\frac{3}{2}{\dot{R}}^{2}\left(1-\frac{\dot{R}}{3c}\right)}{R_{b}\left(1-\frac{\dot{R}}{c}\right)},$$

discretizing the equation obtains

(71) $$\left\{\begin{array}{c} R_{n+1}=R_{n}+dt\cdot \dot{R}=R_{n}+dt\cdot f\left(t,R_{n},{\dot{R}}_{n}\right)\\ {\dot{R}}_{n+1}={\dot{R}}_{n}+dt\cdot \frac{\frac{1}{\rho_{l}}\left(1+\frac{{\dot{R}}_{n}}{c}+\frac{R_{n}}{c}\frac{d}{\mathrm{dt}}\right)\left(P_{B}-P\right)-\frac{3}{2}{\dot{R}}_{n}^{2}\left(1-\frac{{\dot{R}}_{n}}{3c}\right)}{R_{n}\left(1-\frac{{\dot{R}}_{n}}{c}\right)}={\dot{R}}_{n}+dt\cdot g\left(t,R_{n},{\dot{R}}_{n}\right) \end{array}\right),$$

where *f*(*t*,*R_n_*,$\dot{R}$ *_n_*) and *g*(*t*,*R_n_*,$\dot{R}$ *_n_*) are time-varying functions of $\dot{R}$ and $\ddot{R}$, which can be obtained through iteration:

(72) $$\left[\begin{array}{c} R_{n+1}\\ {\dot{R}}_{n+1} \end{array}\right]=\left[\begin{array}{c} R_{n}+\frac{\mathrm{dt}}{6}\cdot \left(k_{1\dot{R}}+2k_{2\dot{R}}+2k_{3\dot{R}}+k_{4\dot{R}}\right)\\ {\dot{R}}_{n}+\frac{\mathrm{dt}}{6}\cdot \left(k_{1\ddot{R}}+2k_{2\ddot{R}}+2k_{3\ddot{R}}+k_{4\ddot{R}}\right) \end{array}\right],$$

and the formula for solving each coefficient is

(73) $$\begin{array}{c} \left[\begin{array}{c} k_{1\dot{R}}\\ k_{1\ddot{R}} \end{array}\right]=\left[\begin{array}{c} f\left(t,R_{n},{\dot{R}}_{n}\right)\\ g\left(t,R_{n},{\dot{R}}_{n}\right) \end{array}\right],\\ \left[\begin{array}{c} k_{2\dot{R}}\\ k_{2\ddot{R}} \end{array}\right]=\left[\begin{array}{c} f\left(t+\frac{t_{0}}{2},R_{n}+\frac{t_{0}}{2}k_{1\dot{R}},{\dot{R}}_{n}+\frac{t_{0}}{2}k_{1\ddot{R}}\right)\\ g\left(t+\frac{t_{0}}{2},R_{n}+\frac{t_{0}}{2}k_{1\dot{R}},{\dot{R}}_{n}+\frac{t_{0}}{2}k_{1\ddot{R}}\right) \end{array}\right],\\ \left[\begin{array}{c} k_{3\dot{R}}\\ k_{3\ddot{R}} \end{array}\right]=\left[\begin{array}{c} f\left(t+\frac{t_{0}}{2},R_{n}+\frac{t_{0}}{2}k_{2\dot{R}},{\dot{R}}_{n}+\frac{t_{0}}{2}k_{2\ddot{R}}\right)\\ g\left(t+\frac{t_{0}}{2},R_{n}+\frac{t_{0}}{2}k_{2\dot{R}},{\dot{R}}_{n}+\frac{t_{0}}{2}k_{2\ddot{R}}\right) \end{array}\right],\\ \left[\begin{array}{c} k_{4\dot{R}}\\ k_{4\ddot{R}} \end{array}\right]=\left[\begin{array}{c} f\left(t+\frac{t_{0}}{2},R_{n}+\frac{t_{0}}{2}k_{3\dot{R}},{\dot{R}}_{n}+\frac{t_{0}}{2}k_{3\ddot{R}}\right)\\ g\left(t+\frac{t_{0}}{2},R_{n}+\frac{t_{0}}{2}k_{3\dot{R}},{\dot{R}}_{n}+\frac{t_{0}}{2}k_{3\ddot{R}}\right) \end{array}\right]. \end{array}$$

## 3 Results

This section conducts numerical calculation under different pressure, temperature and ultrasonic wave frequency for liquid oxygen and methane based on theories proposed in section 2. We analyze the heat transfer mechanism under two specific scenarios: dual-bubble system and high-pressure environment. The gas composition is helium, which is the most commonly used pressurized gas, and liquid vapour. Gas properties, calculaiton methods for proportion of ideal gas and vapour are physical parameters of fluids at working conditions are listed in Table 1 and Table 2.

**Table 1:** Physical parameters of liquid oxygen.

	*ρ* (kg/m3)	*Cv* (kJ/kg)	*Cp* (kJ/kg)	*C* (m/s)	*K* (mW/m-s)	*μ* (μPa-s)
70 K-0.5 bar	1237.0	1.0168	1.6780	1066.5	179.73	371.93
70 K-1.0 bar	1237.1	1.0168	1.6779	1066.6	179.75	372.09
70 K-1.5 bar	1237.2	1.0169	1.6778	1066.8	179.78	372.25
70 K-20 bar	1239.9	1.0201	1.6739	1072.4	180.73	378.22
80 K-0.5 bar	1190.5	0.96976	1.6815	987.51	165.45	261.27
80 K-1.0 bar	1190.6	0.96986	1.6813	987.70	165.48	261.39
80 K-1.5 bar	1190.7	0.96996	1.6812	987.89	165.52	261.51

**Table 2:** Physical parameters of liquid methane.

	*ρ* (kg/m3)	*Cv* (kJ/kg)	*Cp* (kJ/kg)	*C* (m/s)	*k* (mW/m-s)	*μ* (μPa-s)
100 K-1.5 bar	438.97	2.1139	3.4075	1453.0	199.67	151.25
100 K-2.0 bar	439.01	2.1140	3.4071	1453.4	199.72	151.37
100 K-3.0 bar	439.09	2.1143	3.4063	1454.2	199.82	151.62
105 K-1.5 bar	431.99	2.0884	3.4359	1404.7	192.98	134.83
105 K-2.0 bar	432.04	2.0886	3.4354	1405.1	193.04	134.93
105 K-3.0 bar	432.12	2.0888	3.4345	1406.0	193.14	135.13

The integral and differential terms involved in temperature *T_B_* are discretized. The effects of physical parameters, such as ultrasonic wave frequency, amplitude, environment pressure and temperature, on bubble oscillation will be considered. The comparison is conducted between proposed model, adiabatic process, the polytropic coefficients derived by Prosperetti [10], and incompressible model.

### 3.1 Pressure and temperature changes

**Fig. 1:**
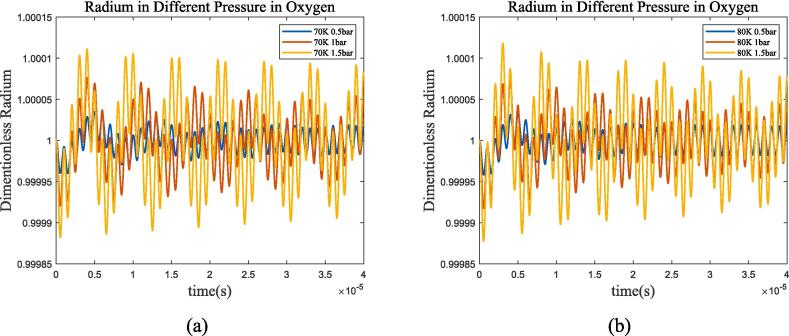
*R*/*R*_0_ of bubble oscillation in liquid oxygen at different pressures and temperatures, with the ultrasonic frequency of 1 MHz. (a) 70 K; (b) 80 K. The blue, red, and yellow lines represent 0.5 bar, 1 bar, and 1.5 bar environment pressure respectively.

**Fig. 2:**
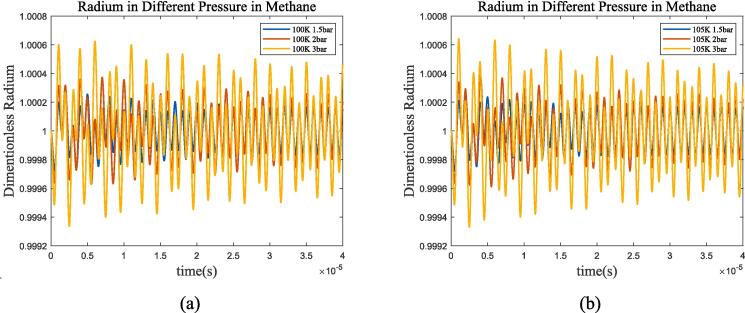
*R*/*R*_0_ of bubble oscillation in liquid methane at different pressures and temperatures, with the ultrasonic frequency of 1 MHz. (a) 100 K; (b) 105 K. The blue, red, and yellow lines represent 1.5 bar, 2 bar, and 3 bar environment pressure respectively.

Firstly, bubble radium is set to 25 μm and ultrasonic frequency is set to 1 MHz to ensure that ultrasonic frequency is much higher than bubble resonance frequency. We perform calculations at different liquid pressure and temperature in liquid methane and oxygen respectively, Fig. 1, Fig. 2 show the radius change in different fluids and operating conditions.

**Fig. 3:**
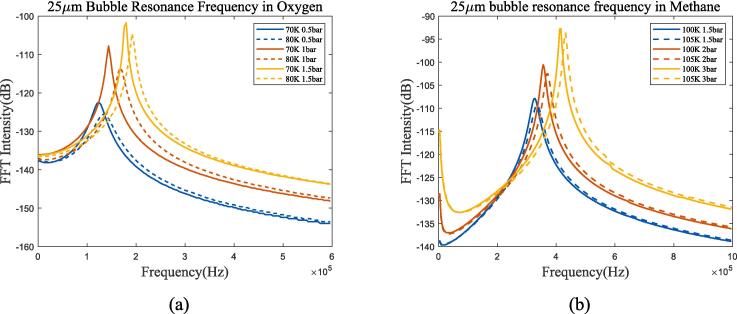
25 μm bubble resonance frequency with different temperature and pressure. (a) liquid oxygen, blue, red, and yellow lines represent 0.5 bar, 1 bar, and 1.5 bar environment pressure, solid and dashed lines represent 70 K and 80 K; (b)liquid methane, blue, red, and yellow lines represent 1.5 bar, 2 bar, and 3 bar environment pressure, solid and dashed lines represent 100 K and 105 K.

The bubble oscillation exhibits a superposition of two mechanisms under ultrasonic excitation: ultrasonic wave driven and resonance in Fig. 1, Fig. 2. After a certain period of time, the resonance component decreases to 0, and the bubble oscillation is completely driven by ultrasonic wave. Fig. 3 selected 150,000 points with intervals of 2.0 × 10^-9 s^ for frequency domain transformation using Fast Fourier Transform(FFT). It can be seen that bubble resonance frequency increases as *P_l_* and temperature increase, for example, it’s approximately 144 kHz, 180 kHz and 192 kHz in liquid oxygen at 70 K-1 bar, 70 K-1.5 bar, and 80 K-1.5 bar.

**Fig. 4:**
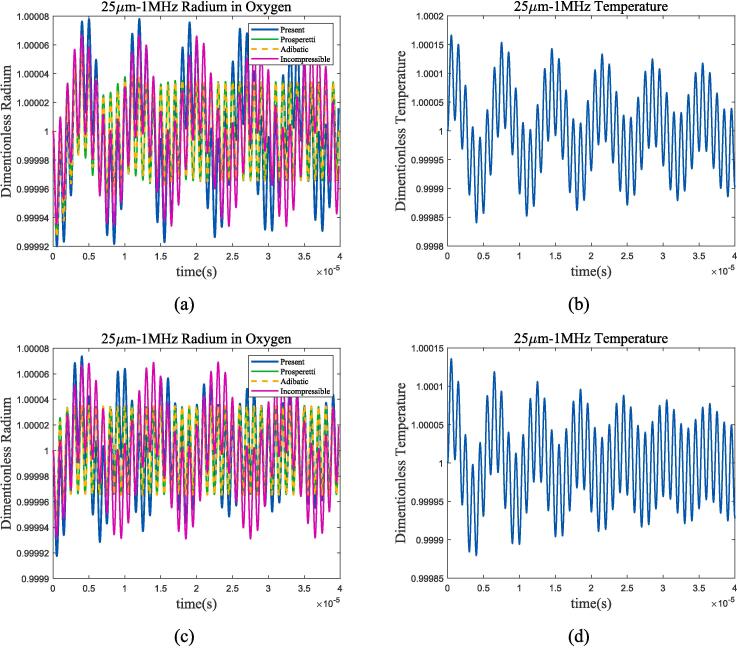
Radium and temperature vibration of bubbles in liquid oxygen, with the liquid pressure of 1 bar; (a)*R*/*R*_0_ at 70 K; (b)*T*/*T*_0_ at 70 K; (c)*R*/*R*_0_ at 80 K; (d)*T*/*T*_0_ at 80 K. Blue represents the method used in this article, green represents Prosperetti’s method, yellow represents adiabatic process, and purple represents incompressible model.

**Fig. 5:**
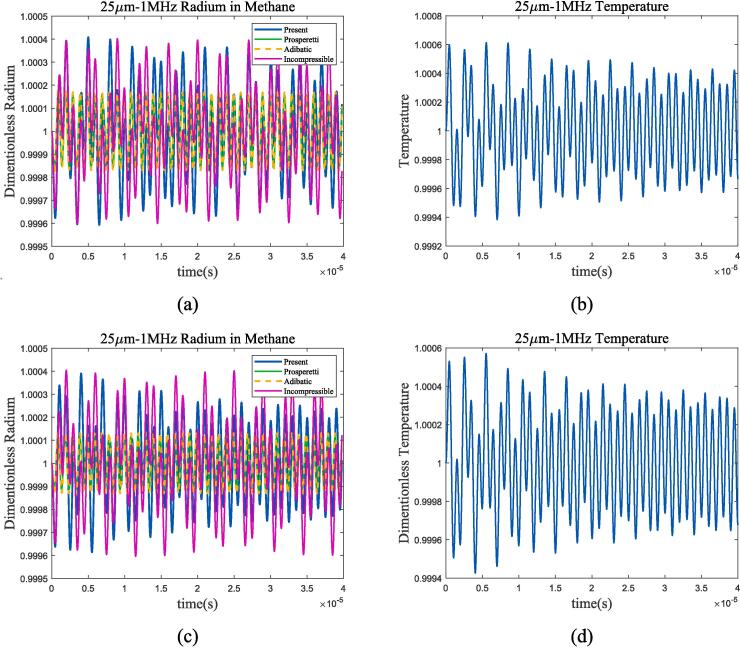
Radium and temperature vibration of bubbles in liquid methane, with the liquid pressure of 2 bar. (a)*R*/*R*_0_ at 100 K; (b)*T*/*T*_0_ at 100 K; (c)*R*/*R*_0_ at 105 K; (b)*T*/*T*_0_ at 105 K. Blue represents the method used in this article, green represents Prosperetti’s method, yellow represents adiabatic process, and purple represents incompressible model.

Take 70 K and 80 K liquid oxygen for analysis at the *P_l_* of 1 bar. Conditions at 100 K, 105 K and 2 bar are chosen for liquid methane. Comparing different thermodynamic mechanisms in selected operating conditions, and calculating the temperature changes in bubble oscillation based on the theory in Section 2 give results as shown in Fig. 4, Fig. 5.

**Fig. 6:**
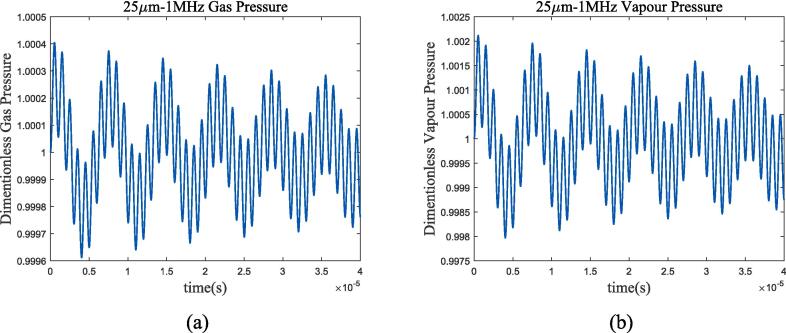
Gas and vapour pressure change during bubble oscillation in 70 K-1 bar liquid oxygen. (a)*P_g_*/*P_g_*_0_; (b)*P_v_*/*P_v_*_0_.

**Fig. 7:**
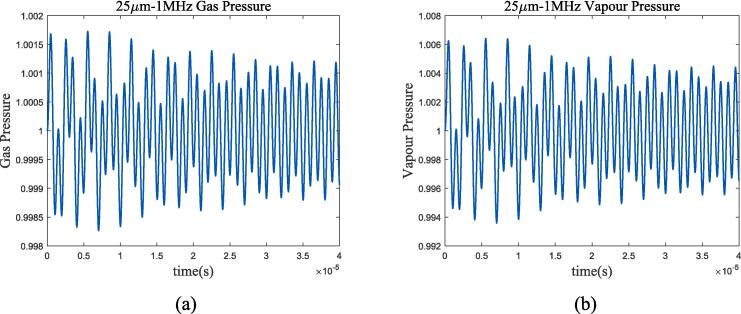
Gas and vapour pressure change during bubble oscillation in 100 K-2 bar liquid methane. (a)*P_g_*/*P_g_*_0_; (b)*P_v_*/*P_v_*_0_.

At the selected ultrasonic frequency, the thermodynamic characteristics of bubbles are significantly different from those of adiabatic process or incompressible model. The temperature change inside the bubble is greater than the method proposed in this article in the adiabatic assumption, resulting in a greater thermal damping that rapidly reduces the resonance component of the bubble and enters the ultrasonic-driven period. Compared to the incompressible model, the thermal damping of the compressible assumption for cryogenic fluids increased as shown in Fig. 4(a) and (c). For further analysis, Fig. 6, Fig. 7 calculate the gas pressure and vapour pressure changes inside bubble in 70 k-1 bar liquid oxygen and 100 k-2 bar liquid methane.

**Fig. 8:**
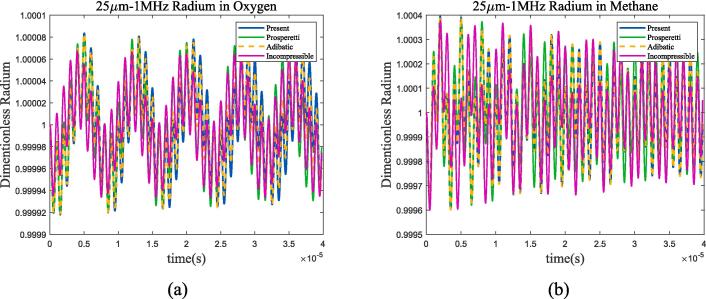
Radium vibration when removing vapour pressure effects. (a)*R*/*R*_0_ in 70 K-1 bar liquid oxygen; (b)*R*/*R*_0_ in 100 K-2 bar liquid methane. Blue represents the method used in this article, green represents Prosperetti’s method, yellow represents adiabatic process, and purple represents incompressible model.

The change in vapour pressure *P_v_* is severer than that that in gas pressure *P_g_*. According to the equation of state, under the assumption of no phase transition, the gas pressure depends on the bubble radium and temperature changes, while the vapour pressure is only determined by temperature. Therefore, it can be considered that the thermal damping caused by temperature change is mainly manifested in the vapour pressure. Assuming the removal of vapour pressure in bubble oscillation, Fig. 8 shows the radium vibration in liquid oxygen and methane.

Obviously, after removing the influence of vapour pressure, the bubble thermodynamic characteristic is very close to adiabatic process. That is, the existence of vapour pressure is the main reason why the bubble heat transfer mechanism differs from adiabatic process, and the two fluids exhibit the same trend, the bubble radium listed in Fig. 8 is almost identical to the adiabatic process.

### 3.2 Linear and nonlinear analysis

**Fig. 9:**
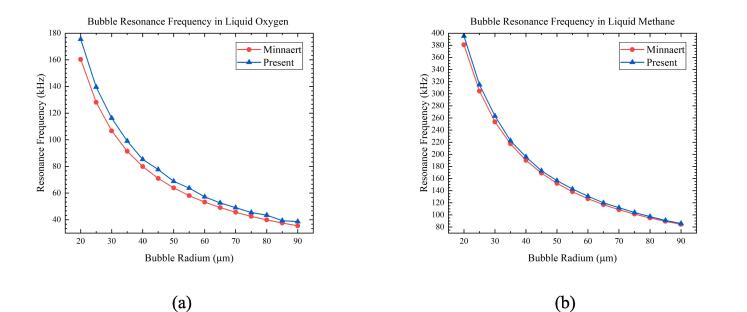
Comparison between Minnaert frequency and cryogenic bubble resonance frequency. (a)70 K-1 bar liquid oxygen; (b)105 K-2 bar liquid methane.

This section will analyze the linear and nonlinear bubble oscillation caused by changes of ultrasonic frequency and amplitude. The 70 K-1 bar liquid oxygen and 100 K-2 bar liquid methane operating conditions were chosen, of which the Minnaert resonance frequency and cryogenic bubble resonance frequency are compared in Fig. 9. Minnaert frequency is calculated by substituting physical properties of mixed gas(helium gas and liquid vapour, in Appendix A) into formula (5), and cryogenic bubble resonance frequency is obtained by numerical calculation.

**Fig. 10:**
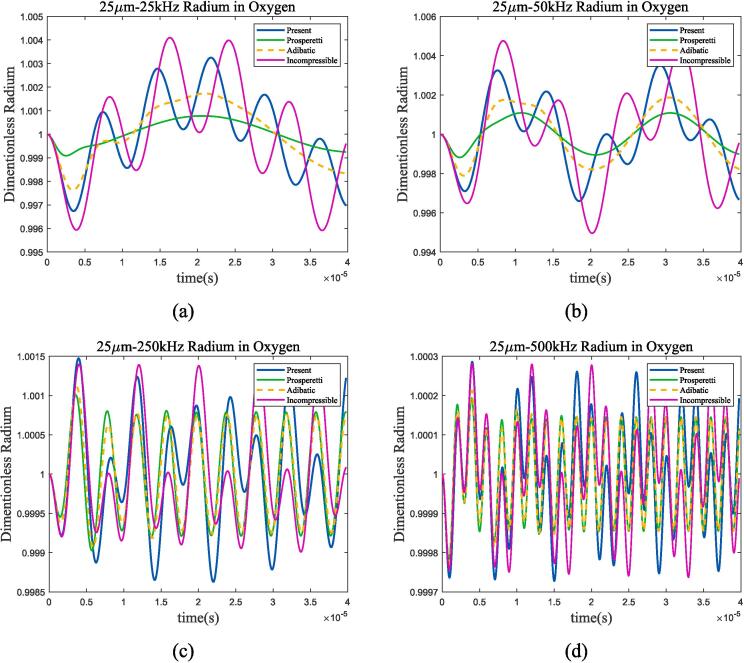
Radium vibration of bubble in 70 K-1 bar liquid oxygen under ultrasonic fields with different frequencies. (a)25 kHz; (b)50 kHz; (c)250 kHz; (d)500 kHz. Blue represents the method used in this article, green represents Prosperetti’s method, yellow represents adiabatic process, and purple represents incompressible model.

**Fig. 11:**
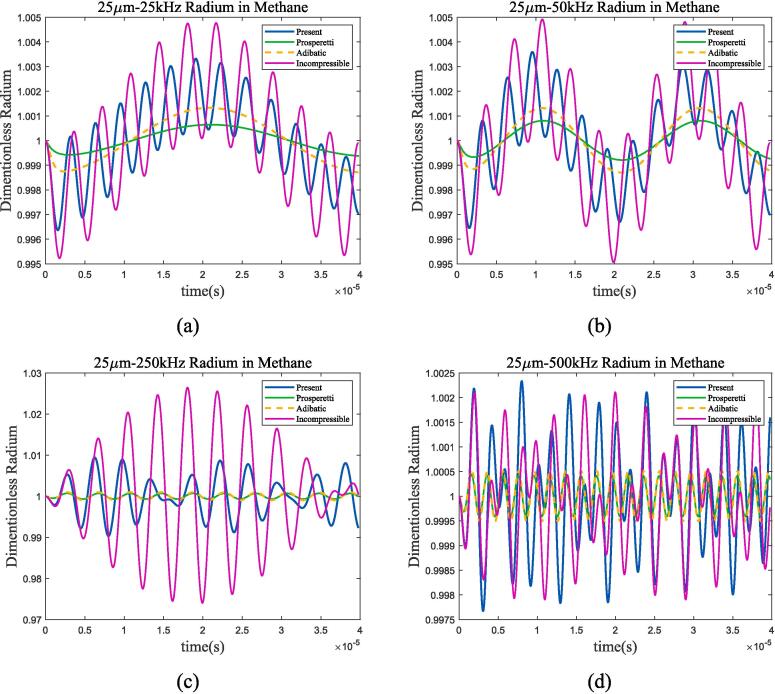
Radium vibration of bubble in 100 K-2 bar liquid methane under ultrasonic fields with different frequencies. (a)25 kHz; (b)50 kHz; (c)500 kHz; (d)1MHz. Blue represents the method used in this article, green represents Prosperetti’s method, yellow represents adiabatic process, and purple represents incompressible model.

**Fig. 12:**
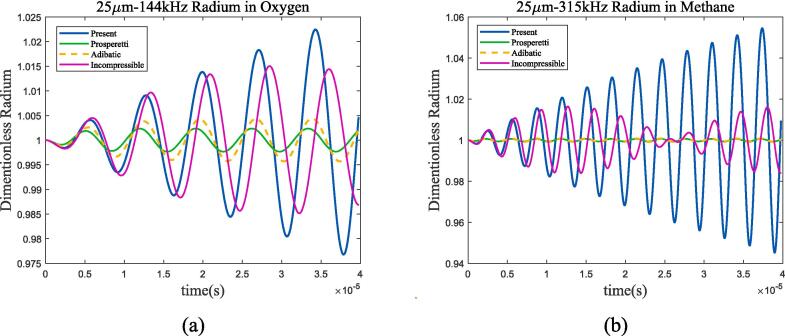
Radium vibration of bubble under ultrasonic fields with resonance frequency. (a)70 K-1 bar liquid oxygen; (b)100 K-2 bar liquid methane. Blue represents the method used in this article, green represents Prosperetti’s method, yellow represents adiabatic process, and purple represents incompressible model.

The larger the bubble radium, the closer its resonance frequency is to the Minnaert frequency. According to Fig. 9(b), it can be seen that the two frequencies are basically equal when the bubble radium is greater than 80um in liquid methane. The bubble radium was set to 25 μm in subsequent analysis, and the resonance frequencies are approximately 144 kHz and 315 kHz in two working conditions. Choosing ultrasonic frequencies of 25 kHz, 50 kHz, 250 kHz, 500 kHz and amplitude of *ε* = 0.01 × *P_l_*, Fig. 10, Fig. 11 show the curve of bubble radium. What’s more, the case when the ultrasonic frequency is equal to the resonance frequency is listed in Fig. 12.

**Fig. 13:**
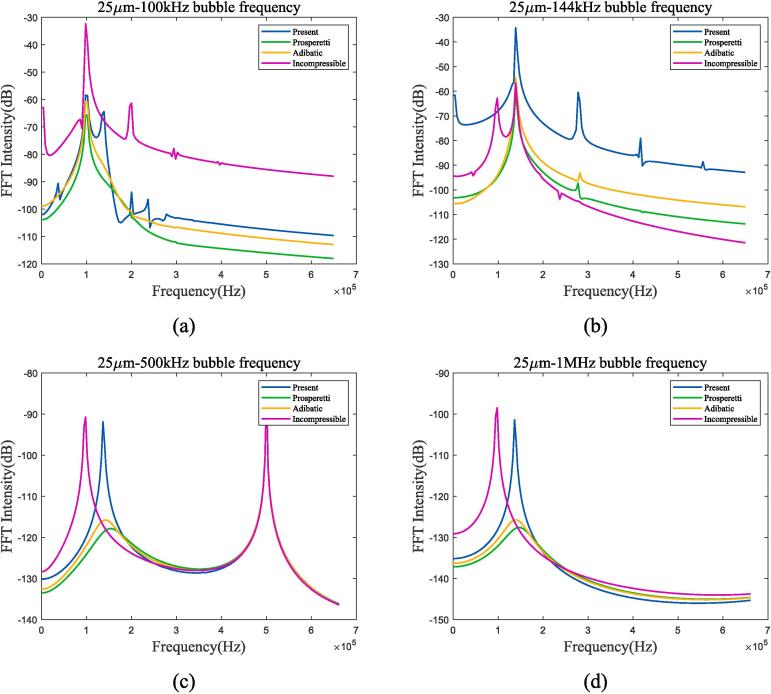
Bubble spectral analysis under different ultrasonic frequency in 70 K-1 bar oxygen. (a) 100 kHz; (b) 144 kHz; (c) 500 kHz; (d) 1 MHz (Remove the excitation frequency of ultrasonic wave). Blue represents the method used in this article, green represents Prosperetti’s method, yellow represents adiabatic process, and purple represents incompressible model.

As shown in the figure, when the frequency of the incident ultrasonic wave approaches the resonance frequency, the amplitude of bubble oscillation significantly increases and reaches its maximum when the two are equal. By the four selected frequency points and the 1 MHz frequency calculated in section 3.1, it can be found that when the ultrasonic frequency is low, the heat transfer between gas and liquid approaches adiabatic process; as the frequency increases, the difference between the two mechanisms becomes larger. Take liquid oxygen as example, Fig. 13 shows the spectral analysis under 100 kHz, 144 kHz, 500 kHz and 1 MHz ultrasonic excitation.

**Fig. 14:**
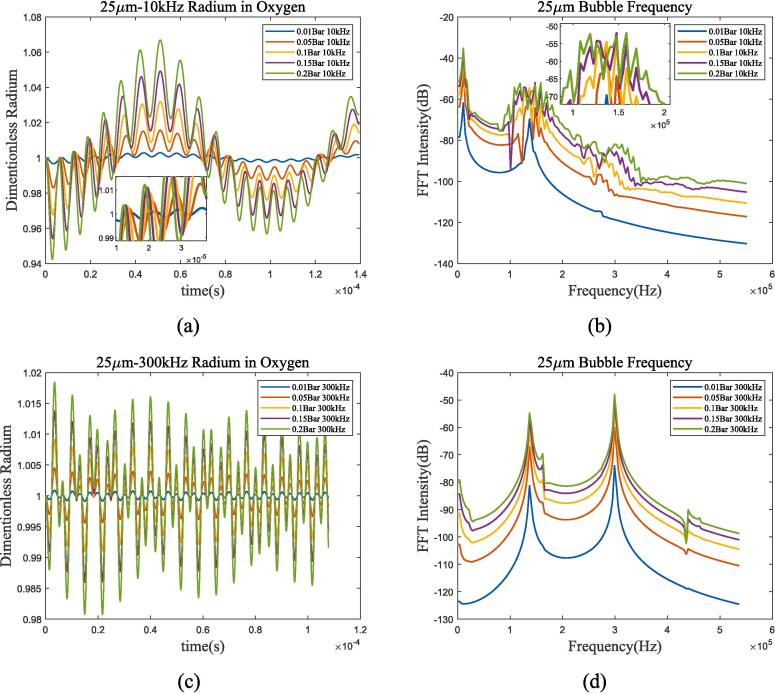
Radium and spectrum of 25 μm bubble under ultrasonic fields with different frequency and amplitude in 70 K-1 bar oxygen. (a)Radium vibration under 10 kHz, 0.01*P_l_*-0.2*P_l_* excitation; (b)Spectrum under 10 kHz, 0.01*P_l_*-0.2*P_l_* excitation; (c) Radium vibration under 300 kHz, 0.01*P_l_*-0.2*P_l_* excitation; (d) Spectrum under 300 kHz, 0.01*P_l_*-0.2*P_l_* excitation.

Under 100 kHz and 144 kHz excitation conditions, bubble oscillation exhibits significant nonlinear effects. As shown in Fig. 13(a) and (b), there are characteristic frequencies other than resonance frequency and ultrasonic frequency. This effect is most pronounced when the frequency of the ultrasonic wave is equal to the resonance frequency, and gradually becomes a linear process as the frequency of the ultrasonic wave continues to increase. The heat transfer mechanism of bubbles has similar characteristic frequencies to that of adiabatic processes and Prosperetti’s methods, but there are differences in intensity, which are determined by the different thermal damping of different mechanisms. There is also a difference in resonance frequency between incompressible and compressible models. To further illustrate the influence of ultrasonic frequency and amplitude on bubble oscillation, Fig. 14 lists the vibration in bubble radium and spectrum when the excitation frequency is 10 kHz and 300 kHz, while the amplitude changes from 0.01*P_l_* to 0.2*P_l_*.

It can be seen that as the ultrasonic amplitude increases, bubble oscillation also changes from linear process to nonlinear process, and the nonlinear characteristics of 10 kHz excitation are more obvious than those of 300 kHz. Taking 10 kHz frequency excitation as an example, when the excitation amplitude is 0.01 bar, bubble oscillation can be regarded as the superposition of resonance and ultrasonic excitation. Increase the amplitude to 0.05 bar, according to Fig. 14(b), there is a harmonic peak in the bubble spectrum, and the number of harmonics increases with increasing amplitude. In contrast, the bubble spectrum excited at 300 kHz is more stable, and a harmonic can be observed at an amplitude of 0.05 bar-0.2 bar.

### 3.3 Bubble-bubble interaction

**Fig. 15:**
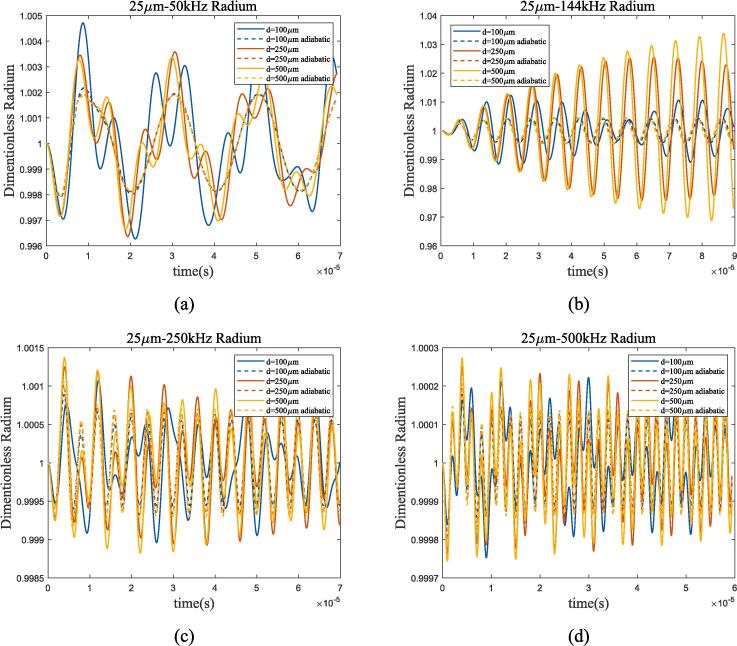
Radium vibration of 25 μm dual-bubble in 70 K-1 bar liquid oxygen under ultrasonic fields with different frequencies. (a) 50 kHz; (b) 144 kHz; (c)250 kHz; (d) 500 kHz. Blue, red, and yellow lines represent 100 μm, 250 μm, and 1.5 mm bubble distance, solid and dashed lines represent proposed method and adiabatic process.

**Fig. 16:**
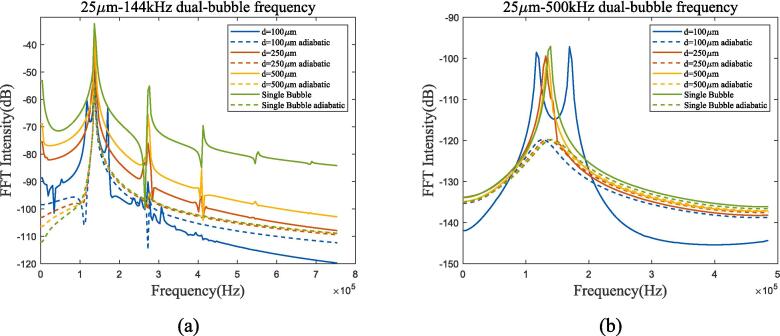
Bubble spectral analysis of 25 μm dual-bubble in 70 K-1 bar liquid oxygen under ultrasonic fields with different frequencies. (a) 144 kHz; (b) 500 kHz (Remove the excitation frequency of ultrasonic wave). Blue, red, yellow and green lines represent 100 μm, 250 μm, 500 μm bubble distance and single bubble, solid and dashed lines represent proposed method and adiabatic process.

This subsection investigates bubble thermodynamic characteristics in a dual-bubble system considering bubble–bubble interaction. Take 70 K-1 bar liquid oxygen as example, a dual-bubble system consisting of two 50 μm radium bubbles is established in Fig. 15, in which the effects of ultrasonic frequency, bubble distance and heat transfer are calculated. Fig. 16 compares the spectral analysis results of different bubble distance under 144 kHz and 500 kHz ultrasonic excitation.

From Fig. 15, Fig. 16, compared to single bubble, the bubble–bubble interaction changes the resonance frequency of the bubbles. The influence of interaction forces on bubbles increases as the spacing decreases. As shown in Fig. 15(b) and Fig. 16, the resonance frequency of 25 μm dual-bubbles with 100 μm spacing changes, while the vibration of bubble radium with 500 μm spacing approaches that of a single bubble. For different spacing, the bubble amplitude still reaches its maximum when the excitation frequency is equal to the resonance frequency.

From the perspective of heat transfer, the resonance frequency of bubble is similar to that of adiabatic process, and the main difference is the resonance intensity, which is the same as the analysis in subsection 3.2. Bubble oscillation exhibits nonlinear characteristics in two cases, namely, low bubble spacing and low ultrasonic frequency excitation. When the bubble spacing or ultrasonic frequency increases, bubble oscillation becomes a linear superposition of ultrasonic excitation and resonance.

### 3.4 High pressure environment

**Fig. 17:**
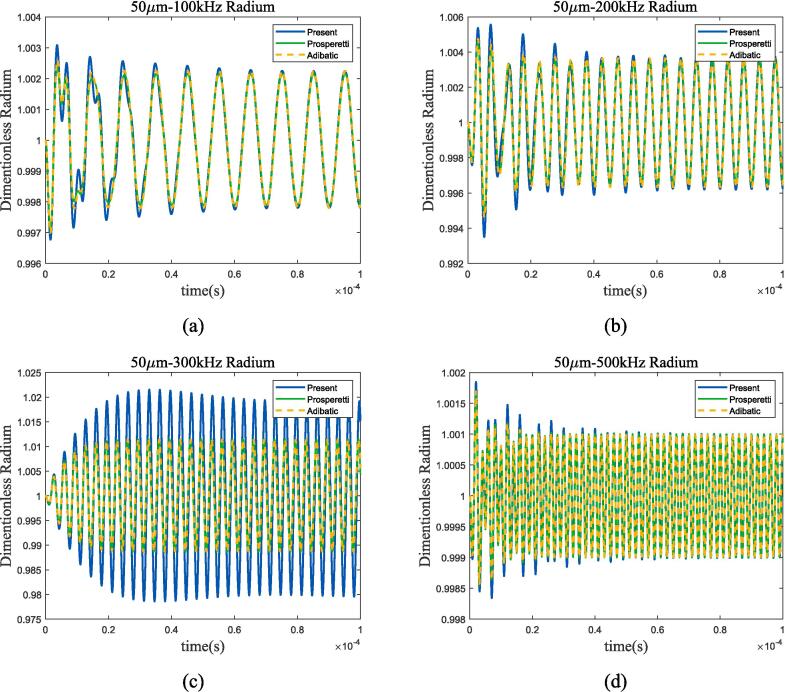
Radium vibration of 50 μm bubble in 70 K-20 bar liquid oxygen under ultrasonic fields with different frequencies. (a) 100 kHz; (b) 200 kHz; (c) 300 kHz; (d) 500 kHz. Blue represents the method used in this article, green represents Prosperetti’s method, yellow represents adiabatic process.

Cryogenic fluids are usually stored in high-pressure environments in practical scenarios, so it is necessary to investigate the influence of high-pressure environments on the bubble thermodynamic. Due to the significant differences between the bubble thermodynamic mechanism and incompressible model in the above analysis, this subsection only compares the proposed method with adiabatic process. Set the environmental pressure *P_l_* to 20 bar and the ultrasonic amplitude to *ε* = 0.01 × *P_l_*, Fig. 17 shows the 50 μm bubble oscillation under 100 kHz, 200 kHz, 300 kHz and 500 kHz ultrasonic excitation.

**Fig. 18:**
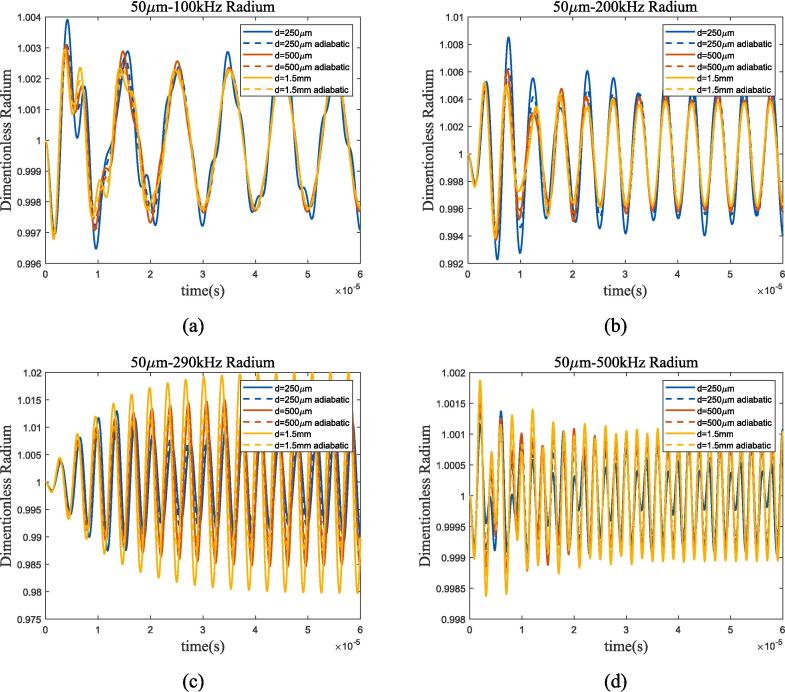
Radium vibration of 50 μm dual-bubble in 70 K-20 bar liquid oxygen under ultrasonic fields with different frequencies. (a) 100 kHz; (b) 200 kHz; (c) 290 kHz; (d)500 kHz. Blue, red, and yellow lines represent 250 μm, 500 μm, and 1.5 mm bubble distance, solid and dashed lines represent proposed method and adiabatic process.

**Fig. 19:**
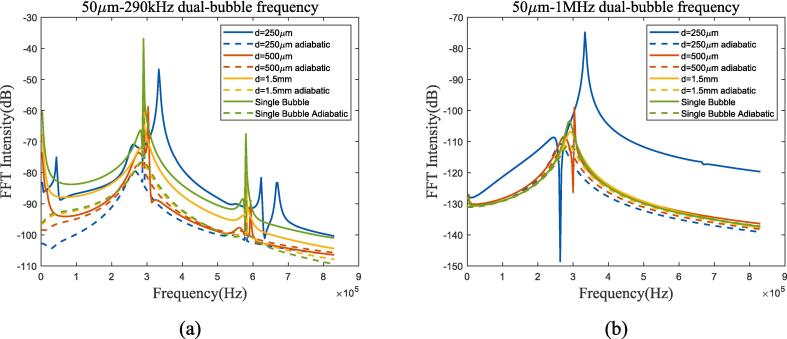
Bubble spectral analysis of 50 μm dual-bubble in 70 K-20 bar liquid oxygen under ultrasonic fields with different frequencies. (a) 290 kHz; (b) 1 MHz (Remove the excitation frequency of ultrasonic wave). Blue, red, yellow and green lines represent 250 μm, 500 μm, 1.5 mm bubble distance and single bubble, solid and dashed lines represent proposed method and adiabatic process.

According to the Minnaert formula, the resonant frequency of 50 μm bubble is approximately 290 kHz. When the frequency of acoustic wave approaches the resonance frequency, bubble still have obvious resonance characteristics, and differs greatly from adiabatic process; as the frequency moves away from the resonant frequency, the heat transfer mechanism of bubble is almost identical to that of adiabatic processes. On this basis, a 50 μm dual-bubble analysis was conducted, and Fig. 18, Fig. 19 show the radius variation and spectrum analysis.

Compared to 1 bar environment pressure in subsection 3.3, bubble spacing has a similar effect while bubble oscillation almost coincides with the adiabatic process, apart from the condition that ultrasonic frequency being equal to the resonance frequency(Fig. 18(c) and Fig. 19(a)), and spectrum analysis also proves it. The spectrum in Fig. 19(b) shows that under high-pressure conditions, high-frequency ultrasonic excitation can also maintain linear characteristics. The variation trends of bubble oscillation in linear, nonlinear, and heat transfer are the same as those in low-pressure environments. In summary, the bubble thermodynamic mechanism in high-pressure environments can be approximated as an adiabatic linear mechanism when ultrasonic frequency is much higher than resonance frequency.

## 4 Conclusion

This article focuses on the bubble thermodynamic under ultrasonic excitation in cryogenic fluids. Assuming that the gas inside the bubble consists of ideal gas and vapour, a theoretical derivation of temperature changes during bubble oscillation is conducted based on the energy equation. The radium variation of bubble in liquid oxygen and liquid methane was obtained through numerical calculations, and its heat transfer mechanism was analyzed. The following conclusions were drawn:

Firstly, the presence of vapour pressure significantly increases the thermal damping of bubble, the heat transfer mechanism between gas and liquid is between isothermal and adiabatic process. The bubble oscillation is close to the adiabatic process after removing the effect of vapour pressure. Compared to the incompressible model, the compressibility assumption of cryogenic fluids changes the resonance frequency and thermal damping of bubbles.

Secondly, the heat transfer mechanism is influenced by the relationship between ultrasonic excitation frequency and bubble resonance frequency. When the frequency of the excited ultrasonic wave is lower than the resonance frequency, the bubble oscillation has obvious nonlinear characteristics. As the ultrasonic frequency gradually increases to much higher than the resonance frequency, bubble oscillation transforms into linear process. Spectral analysis shows that bubbles and adiabatic processes have similar resonance frequencies, where the difference mainly reflects in the strength difference caused by thermal damping.

Thirdly, a dual-bubble system dominated by the second Bjerknes force was comprehensively analyzed. As the same in a single bubble, the thermodynamic mechanism of double-bubble has a similar resonance frequency to the adiabatic process, but with different intensities. The nonlinear effects are mainly reflected in low-frequency ultrasonic excitation and low bubble spacing. Finally, through the analysis of bubble thermal dynamics in high-pressure environments, it is believed that the heat transfer mechanism of bubbles can be approximated as adiabatic process in high-pressure environments.

Due to the complexity of bubble thermodynamic mechanisms, this article briefly analyzes their characteristics through numerical calculation. Furthermore, it is difficult to obtain accurate analytical solutions for relevant linearization parameters, such as resonance frequency, damping coefficient, etc. Future researches will focus on the linear and nonlinear effects of bubble thermodynamics in cryogenic fluids, including heat transfer mechanisms and multi-bubble interactions. Some machine learning related methods may be able to fit these parameters, providing a basis for bubble measurement in cryogenic fluids.

## CRediT authorship contribution statement

**Jin Zhang:** Writing – original draft, Methodology, Formal analysis. **Yu Zhang:** Supervision, Investigation, Funding acquisition. **Yong Chen:** Writing – review & editing, Funding acquisition, Conceptualization. **Xiaobo Rui:** Writing – review & editing, Formal analysis, Data curation. **Yao Yu:** Resources. **Yu Wu:** Supervision, Investigation, Funding acquisition. **Jie Yang:** Visualization. **Lei Qi:** Funding acquisition.

## Declaration of competing interest

The authors declare that they have no known competing financial interests or personal relationships that could have appeared to influence the work reported in this paper.

## Figures and Tables

**Table 3:** Physical properties of gas helium.

	*ρ* (kg/m3)	*Cv* (kJ/kg)	*Cp* (kJ/kg)	*k* (mW/m-K)	Compact Factor
70 K-0.5 bar	0.34698	3.1164	5.1952	58.107	1.0009
70 K-1.0 bar	0.69332	3.1169	5.1972	58.150	1.0018
70 K-1.5 bar	1.0390	3.1173	5.1992	58.196	1.0028
70 K-20 bar	13.391	3.1351	5.2664	60.726	1.0361
80 K-0.5 bar	0.30363	3.1163	5.1947	63.477	1.0008
80 K-1.0 bar	0.60675	3.1168	5.1962	63.519	1.0017
80 K-1.5 bar	0.90936	3.1172	5.1977	63.564	1.0025
100 K-1.5 bar	0.72777	3.1170	5.1959	73.757	1.0021
100 K-2.0 bar	0.96968	3.1174	5.1968	73.801	1.0028
100 K-3.0 bar	1.4525	3.1181	5.1986	73.892	1.0043
105 K-1.5 bar	0.69318	3.1170	5.1956	76.209	1.0021
105 K-2.0 bar	0.92361	3.1173	5.1964	76.253	1.0027
105 K-3.0 bar	1.3835	3.1180	5.1980	76.334	1.0041

**Table 4:** Physical propertiess of liquid vapour.

	*ρ* (kg/m3)	*Cv* (kJ/kg)	*Cp* (kJ/kg)	*k* (mW/m-K)
70 K-Oxygen	0.34573	1.0167	1.6781	5.9925
80 K-Oxygen	1.4684	0.96972	1.6816	7.0277
100 K-Methane	0.67457	2.1136	3.4084	10.528
105 K-Methane	1.0613	2.0881	3.4367	11.188

**Table 5:** Coefficients in ancillary function of liquid vapour.

	Methane		Oxygen	
*i*	*Ni*	*ki*	*Ni*	*ki*
1	−6.036219	1	−6.0595	1
2	1.409353	1.5	1.305	1.5
3	−0.4945199	2	−0.54178	2.2
4	−1.443048	4.5	−1.941	4.8
5			0.35514	6.2

## Data Availability

Data will be made available on request.

## Appendix B Solution of auxiliary function *Hk*

Equation (41) is auxiliary function to solve the temperature gradient inside bubble. Variables *R* and *T_B_* are regarded as functions with dependent variable *t*, thus Equation (41) is a first order linear non-homogeneous differential equation. Another form of Equation (41) is

(B1) $$\frac{dH_{k}}{H_{k}}=\frac{1}{H_{k}}\left(\frac{P_{c}}{\rho_{g}R_{b}}\dot{R}+\frac{C_{\mathrm{vg}}}{3}{\dot{T}}_{B}\right)dt-\left[\frac{9{\left(2k+1\right)}^{2}\pi^{2}\rho_{g}k_{g}}{4C_{\mathrm{vg}}}\right]R_{b}dt,$$

Iintegrating *t* on both sides simultaneously yields

(B2) $$\ln H_{k}=\int \frac{1}{H_{k}}\left(\frac{P_{c}}{\rho_{g}R_{b}}\dot{R}+\frac{C_{\mathrm{vg}}}{3}{\dot{T}}_{B}\right)dt-\int \left[\frac{9{\left(2k+1\right)}^{2}\pi^{2}\rho_{g}k_{g}}{4C_{\mathrm{vg}}}\right]R_{b}dt+\ln C_{2},$$

*C*2 is constant. Equation (B2) can be written in exponential form as

(B3) $$H_{k}=C_{2}e^{\int \frac{1}{H_{k}}\left(\frac{P_{c}}{\rho_{g}R_{b}}\dot{R}+\frac{C_{\mathrm{vg}}}{3}{\dot{T}}_{B}\right)dt}\cdot e^{-\int \left[\frac{9{\left(2k+1\right)}^{2}\pi^{2}\rho_{g}k_{g}}{4C_{\mathrm{vg}}}\right]R_{b}dt}.$$

To calculate the solution of *C*2, a new formula is introduced

(B4) $$C_{3}\left(t\right)=C_{2}e^{\int \frac{1}{H_{k}}\left(\frac{P_{c}}{\rho_{g}R_{b}}\dot{R}+\frac{C_{\mathrm{vg}}}{3}{\dot{T}}_{B}\right)dt},$$

so the derivative of *Hk* is

(B5) $${\dot{H}}_{k}={\dot{C}}_{3}\left(t\right)\cdot e^{-\int \left[\frac{9{\left(2k+1\right)}^{2}\pi^{2}\rho_{g}k_{g}}{4C_{\mathrm{vg}}}\right]R_{b}dt}-\left[\frac{9{\left(2k+1\right)}^{2}\pi^{2}\rho_{g}k_{g}}{4C_{\mathrm{vg}}}\right]R_{b}e^{-\int \left[\frac{9{\left(2k+1\right)}^{2}\pi^{2}\rho_{g}k_{g}}{4C_{\mathrm{vg}}}\right]R_{b}dt},$$

substitute formula (B5) into the original equation (41), the solution of *C*3 is

(B6) $$C_{3}\left(t\right)=\int \left(\frac{P_{c}}{\rho_{g}R_{b}}\dot{R}+\frac{C_{\mathrm{vg}}}{3}{\dot{T}}_{B}\right)e^{\int \left[\frac{9{\left(2k+1\right)}^{2}\pi^{2}\rho_{g}k_{g}}{4C_{\mathrm{vg}}}\right]R_{b}dt}+C_{4},$$

according to formula (B3), (B4) and (B6), we can get

(B7) $$H_{k}=e^{-\int \left[\frac{9{\left(2k+1\right)}^{2}\pi^{2}\rho_{g}k_{g}}{4C_{\mathrm{vg}}}\right]R_{b}dt}\left(\int \left(\frac{P_{c}}{\rho_{g}R_{b}}\dot{R}+\frac{C_{\mathrm{vg}}}{3}{\dot{T}}_{B}\right)e^{\int \left[\frac{9{\left(2k+1\right)}^{2}\pi^{2}\rho_{g}k_{g}}{4C_{\mathrm{vg}}}\right]R_{b}dt}+C_{4}\right).$$

*C*_4_ is a constant. According to the analysis in section 2.2, *C*_4_ = 0, so equation (B7) can be expressed as formula (43) and (44) in the article.
